# Tacrolimus Protects Podocytes from Injury in Lupus Nephritis Partly by Stabilizing the Cytoskeleton and Inhibiting Podocyte Apoptosis

**DOI:** 10.1371/journal.pone.0132724

**Published:** 2015-07-10

**Authors:** Ruyi Liao, Qinghua Liu, Zhihua Zheng, Jinjin Fan, Wenxing Peng, Qingyu Kong, Huijuan He, Shicong Yang, Wenfang Chen, Xueqing Tang, Xueqing Yu

**Affiliations:** 1 Department of Nephrology, The First Affiliated Hospital, Sun Yat-sen University, Guangzhou, China; 2 Guangdong Provincial Key Laboratory of Nephrology, Guangzhou, China; 3 Key Laboratory of Nephrology, Ministry of Health, Guangzhou, China; 4 Department of Pathology, The First Affiliated Hospital, Sun Yat-sen University, Guangzhou, China; INSERM-Université Paris-Sud, FRANCE

## Abstract

**Objective:**

Several studies have reported that tacrolimus (TAC) significantly reduced proteinuria in lupus nephritis (LN) patients and mouse models. However, the mechanism for this effect remains undetermined. This study explored the mechanism of how TAC protects podocytes from injury to identify new targets for protecting renal function.

**Methods:**

MRL/lpr mice were given TAC at a dosage of 0.1 mg/kg per day by intragastric administration for 8 weeks. Urine and blood samples were collected. Kidney sections (2μm) were stained with hematoxylin-eosin (HE), periodic acid-Schiff base (PAS) and Masson's trichrome stain. Mouse podocyte cells (MPC5) were treated with TAC and/or TGF-β_1_ for 48h. The mRNA levels and protein expression of synaptopodin and Wilms’ tumor 1 (WT1) were determined by real-time PCR, Western blotting and/or immunofluorescence, respectively. Flow cytometry was used to detect cell apoptosis with annexin V. Podocyte foot processes were observed under transmission electron microscopy. IgG and C3 deposition were assessed with immunofluorescence assays and confocal microscopy.

**Results:**

Synaptopodin expression significantly decreased in MRL/lpr disease control mice, accompanied by increases in 24-h proteinuria, blood urea nitrogen, and serum creatinine. TAC, however, reduced proteinuria, improved renal function, attenuated renal pathology, restored synaptopodin expression and preserved podocyte numbers. In MPC5 cells, TGF-β_1_ enhanced F-actin damage in podocytes and TAC stabilized it. TAC also decreased TGF-β_1_-induced podocyte apoptosis *in vitro* and inhibited foot process fusion in MRL/lpr mice. In addition, our results also showed TAC inhibited glomerular deposition of IgG and C3.

**Conclusion:**

This study demonstrated that TAC reduced proteinuria and preserved renal function in LN through protecting podocytes from injury partly by stabilizing podocyte actin cytoskeleton and inhibiting podocyte apoptosis.

## Introduction

Lupus nephritis (LN) is a major cause of morbidity and mortality in patients with systemic lupus erythematosus (SLE). Proteinuria is an important risk factor for the progression of renal diseases in patients with LN [1]. A recent review reported that tacrolimus (TAC), a calcineurin inhibitor (CNI), was able to reduce proteinuria and prevent the progression of the nephropathy in lupus mice or LN patients [2]. Our previous clinical trial also demonstrated that TAC treatment resulted in a quick reduction of proteinuria, and remission of LN [3]. However, the precise mechanisms of mediating the anti-albuminuric effects of TAC are still quite poorly understood. Notably, a previous study showed that cyclosporin A (CsA), another CNI, blocks the calcineurin-mediated dephosphorylation of synaptopodin, which in turn, protects synaptopodin from cathepsin L-mediated degradation, thereby maintaining the integrity of the glomerular filtration barrier and safeguarding against proteinuria [4]. The aim of this study was to investigate the mechanisms of TAC effects on anti-albuminuria and protection of renal function, which may provide a potential new way to treat LN.

## Materials and Methods

### Animal models of lupus nephritis and normal controls

MRL/lpr mice, an established model of LN, were chosen as the animal model for this study. Female MRL/lpr mice (n = 30) weighing 16 to 20g at 12 weeks old were obtained from Academia Sinica Shanghai Institute of Pharmaceutical Research and were specific pathogen free (SPF) grade. Age and weight matched SPF female C57BL/6 mice (n = 18) obtained from Sun Yat-sen University Animal Center were used as normal control (NC). MRL/lpr mice were randomly divided into disease control group (DC, 10 mice for week zero and eight, respectively) and TAC treatment group (TAC, 10 mice for week eight). C57BL/6 mice were randomly divided into NC week zero and eight. Mice from the treatment group were given TAC at a dosage of 0.1 mg/kg per day by intragastric administration for 8 weeks. Control groups (including the NC and DC groups) received daily intragastric administration of equal amounts of saline. All mice were anesthetized with isoflurane and sacrificed via cervical dislocation. Animal protocols and procedures were approved by the Animal Care and Use Committee of Sun Yat-sen University and complied with appropriate institutional regulations.

### Sample collection and analysis

Urine samples were collected in metabolic cages to examine the levels of 24-h urinary protein excretion and ratios of urinary protein to creatinine. Blood samples were obtained by eye puncture under ether anesthesia to examine the levels of BUN and serum creatinine at 0 and 8 weeks as the mice were sacrificed. A coronal slice of the kidney was removed from each mouse, fixed in 10% neutral-buffered formalin, and embedded in paraffin. Some kidney samples also were snap-frozen in liquid nitrogen prior to storage at -80°C, and a small portion was fixed in 2–4% glutaric dialdehyde for transmission electron microscopy. Immunofluorescence and Western blot analyses were conducted to observe protein distribution and levels and real time-PCR was performed to measure mRNA contents.

### Pathologic and digital image analysis

At the end of the eight-week treatment period, kidney tissues were immersion-fixed in 4% paraformaldehyde/phosphate-buffered saline and embedded in paraffin. Sections (2 μm) were stained with hematoxylin-eosin (HE), periodic acid-Schiff base (PAS) and Masson's trichrome stain, and images of the section were captured at 400× magnification using a Zeiss Axioplan microscope equipped with a Sony DXC-950P 3CCD color camera (Sony Corporation; Tokyo, Japan) and further analyzed using KS-400 image analysis software (Windows version 3.0; Carl Zeiss Vision; Oberkochen, Germany). Thirty glomeruli for each kidney section were digitally quantified. Pathological scores of each mouse were calculated according to the glomerular, renal tubular and pathology rating criteria described previously [5].

### Antibodies

Rabbit-anti-Wilms’ tumor 1 (WT1) antibody was obtained from Abcam (Hong Kong, China) and mouse-anti-synaptopodin antibody was obtained from Acris Biotechnology (Herford, Germany). Fluorescein isothiocyanate (FITC)-conjugated goat anti-rabbit IgG antibody and goat anti-mouse IgG antibody were obtained from Sigma Chemical Co. (St. Louis, MO, USA). FITC-conjugated rabbit anti-mouse IgG antibody and rabbit anti-mouse complement C3 antibody were obtained from Beijing Biosynthesis Biotechnology Co. LTD. (Beijing, China).

### Immunofluorescence and podocyte counting

Five-micrometer cryostat sections were cut, transferred to Starfrost slides, air dried, and stored at -20°C until used to explore immune complex deposit. For immunofluorescence, the paraffin slides were cut into 2-μm sections, incubated with the antigen for 3 h at room temperature, then washed in PBS and incubated at 4°C overnight with the primary antibody diluted in 5% BSA in PBS (rabbit anti-WT1, 1:100, mouse anti-synaptopodin, 1:200) and thereafter washed in PBS. The slides were incubated with the FITC-conjugated anti-rabbit IgG antibody and FITC-conjugated anti-mouse IgG antibody (1:1000) for 60 min, washed in PBS, and covered with mounting medium (R&D Systems; Minneapolis, MN, USA). For IgG and C3 staining, frozen sections (5μm) were fixed for minutes in cold (-20°C) acetone and then stained with primary FITC-conjugated rabbit anti-mouse IgG and C3 antibody (1:100), respectively. F-actin and nuclei were stained with phalloidin and DAPI, respectively. For each antibody, all samples were stained in a single session.

Podocyte counting was performed according to the disector/fractionator combination method described previously [6].

### Western blot analysis

Mouse kidney tissue was harvested and lysed with cell lysis buffer (Cell Signaling Technology; Danvers, MA, USA) and proteins were extracted for Western blot analysis. Western blot analysis was performed using standard procedures. Soluble material was subjected to SDS-PAGE with a 7.0% acrylamide gel and transferred to a nitrocellulose membrane (Bio-Rad; Hercules, CA, USA) by electrophoretic transblotting for 90 min using Trans-Blot SD (Bio-Rad). After blocking with BSA, membranes were probed with primary antibody (mouse anti-synaptopodin; Acris; Herford, Germany; 1:1000 dilution) overnight at 4°C. Membranes were then incubated with secondary antibody (anti-rabbit IgG, 1:1000; and anti-mouse- IgG, 1:1000; Cell Signaling Technology; Boston, MA, USA). The signals were scanned and quantified by FluorChem8900 software (AlphaInnotech, Witec; Littau, Switzerland). Following enhanced chemiluminescence detection, the membranes were stripped and proteins rehybridized with anti-β-actin antibody or anti-glyceraldehyde-3-phosphate dehydrogenase (GAPDH) antibody (1:1000; DAKO; Carpinteria, CA, USA). Protein levels were expressed as protein/β-actin or protein/GAPDH ratio to minimize differences in sample loading.

### mRNA isolation, cDNA synthesis, and real-time PCR

Total RNA was extracted from kidney tissue by the Trizol-method and used for cDNA synthesis. For WT1, synaptopodin, and GAPDH, forward and reverse primers (Invitrogen; Carlsbad, CA, USA) were designed using Primer Express 5.0 software (PE Applied Biosystems; Foster City, CA, USA). The sequences of the primers are shown in Table 1. Real-time PCR was performed using the ABI PRISM 7900 sequence detector and software (Applied Biosystems). All measurements were performed in duplicate. Amplification cycles were 95°C for 10 min, followed by 40 cycles at 95°C for 30 s, 72°C for 60 s, and 55°C for 30 s. To correct for the amount of tissue used for RNA extraction and the efficiency of cDNA synthesis, we used the ratio between the mRNA levels of WT1 and synaptopodin and the mRNA level of GAPDH, a constitutively expressed gene. The suitability of GAPDH as a housekeeping gene for standardization of mRNA levels was confirmed by testing correlations between GAPDH mRNA for all samples (each group *n* = 6).

**Table 1:** cDNA primers used in real-time PCR measurements.

	Primer Sequence
**Synaptopodin (270 bp)**	Forward: 5’-CAAGCAGCAGCCATACCAG-3’
	Reverse: 5’-CCGAGGCAGAGC AGG AGAT-3’
**WT1 (139 bp)**	Forward: 5’-AATGGACAGAAGGGCAGAGC-3’
	Reverse: 5’-CTCCAGATACACGCCGCACA-3’
**GAPDH (105 bp)**	Forward: 5’- CATGGCCTTCCGTGTTCCTA-3’
	Reverse: 5’-CCTGCTTCACCACCTTCTTGAT-3’

### Transmission electronic microscopy

Small pieces of renal cortex were fixed in 4% glutaraldehyde and 1% paraformaldehyde, dehydrated, and embedded in Spurr resin. In semi-thin sections stained with toluidine blue, non-sclerotic glomeruli were localized. Ultrathin sections were made of one or two glomeruli per tissue specimen and stained with lead citrate for transmission electron microscopy. Four to ten photographs, covering one or two glomerular cross-sections, were made with a Philips CM10 transmission electron microscope (Philips; Eindhoven, The Netherlands). Images with a final magnification of approximately 13,500× were obtained.

### Mouse podocyte cell (MPC5) culture

Conditionally immortalized differentiated mouse podocyte cells (MPC5 cells) (a kind gift from Prof. Peter Mundel, Mount Sinai School of Medicine, through Prof. Jie Ding, Peking University) were cultured as previously described [7]. Briefly, MPC5 cells proliferated in a 33°C, 5% CO2 cell culture box and differentiated in a 37°C, 5% CO2 cell culture box, respectively. MPC5 cells were grown to 80% confluence, serum-starved for 12 to 18 h, and treated with TAC (10–40 μM) and/or TGF-β_1_ (5–20 ng/ml) for 48 h. Immunofluorescence was used to observe changes in cytoskeleton F-actin and flow cytometry detected cell apoptosis with annexin V.

### Statistical analyses

Data are presented as means ± SEM. For parametric data, one-way ANOVA followed by Bonferonni post hoc analysis were used. For nonparametric data, the Kruskal-Wallis and Mann-Whitney U test were used. *P*<0.05 was considered statistically significant. Analyses were performed with SPSS software, version 19.0 (SPSS, Inc.; Chicago, IL, USA).

## Results

### Tacrolimus reduced proteinuria and preserved renal function in MRL/lpr mice

**Fig 1:**
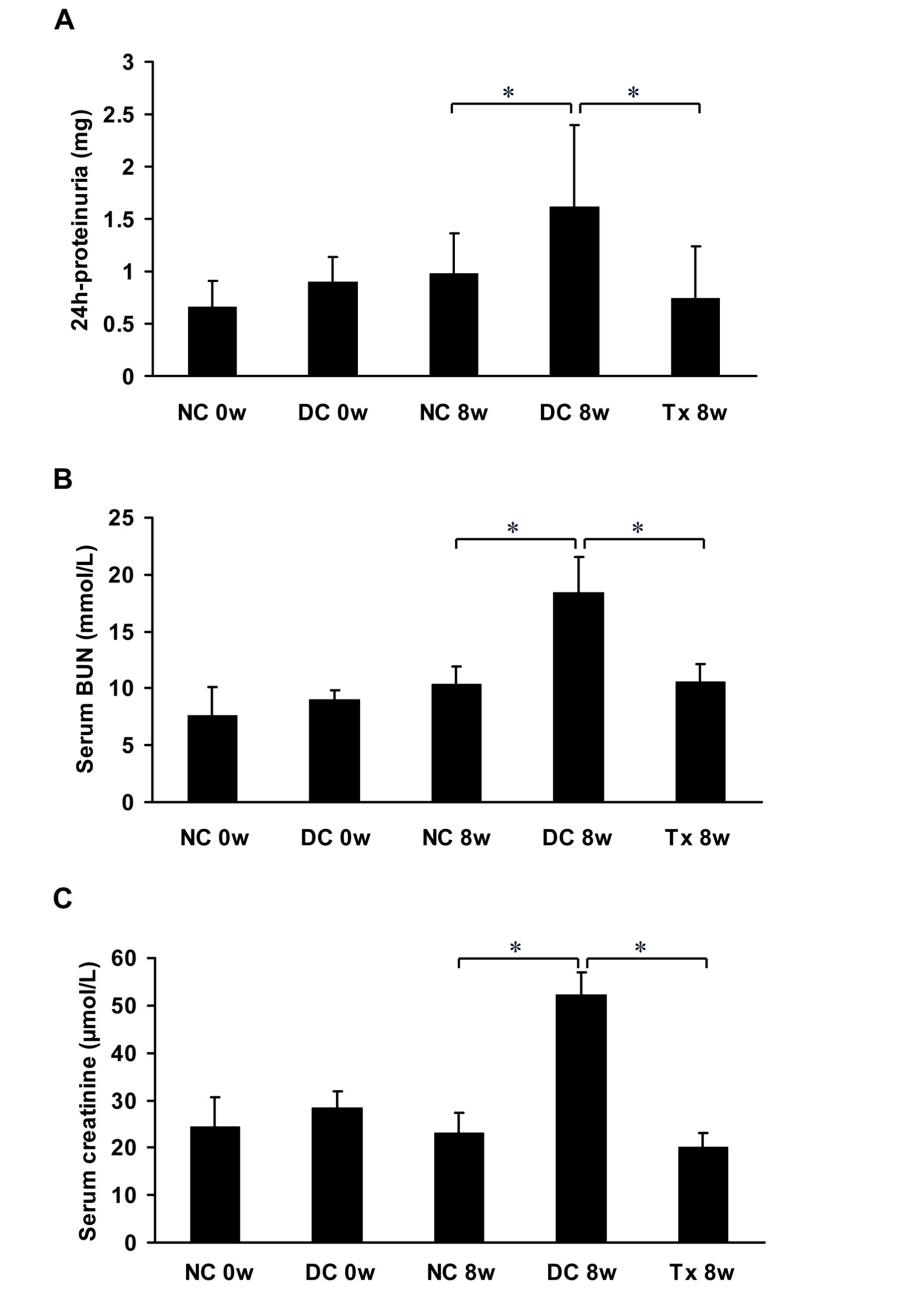
TAC reduced proteinuria and preserved renal function in MRL/lpr mice. (A) All animals (starting at 12 weeks of age, 6 per group) were placed in metabolic cages for urine collection before therapy (0 day) and at the end of the study (8 weeks treatment). Urine samples were used to examine the levels of 24 hour urinary protein excretion. (B, C) Blood samples were obtained from MRL/lpr mice (starting at 12 weeks of age, 6 per group) by eye puncture under ether anesthesia to examine the levels of BUN and serum creatinine just before all mice were killed at 0 week and 8 weeks. NC, normal control group; DC, disease control group; Tx, treatment group. *P < 0.05.

Proteinuria was significantly higher in MRL/lpr mice compared to that in the normal control group, and a significant reduction of proteinuria was observed in the treatment group compared to the disease control group. Differences in paired sets of values after eight weeks of treatment were statistically significant (Fig 1A). The BUN (Fig 1B) and serum creatinine levels (Fig 1C) were significantly higher in the disease control group than those in the normal control group. After TAC treatment, the BUN and serum creatinine levels significantly decreased at 8 weeks compared to the disease control group. Differences in paired sets of values after eight weeks of treatment were statistically significant.

### Tacrolimus attenuated renal pathology in MRL/lpr mice

**Fig 2:**
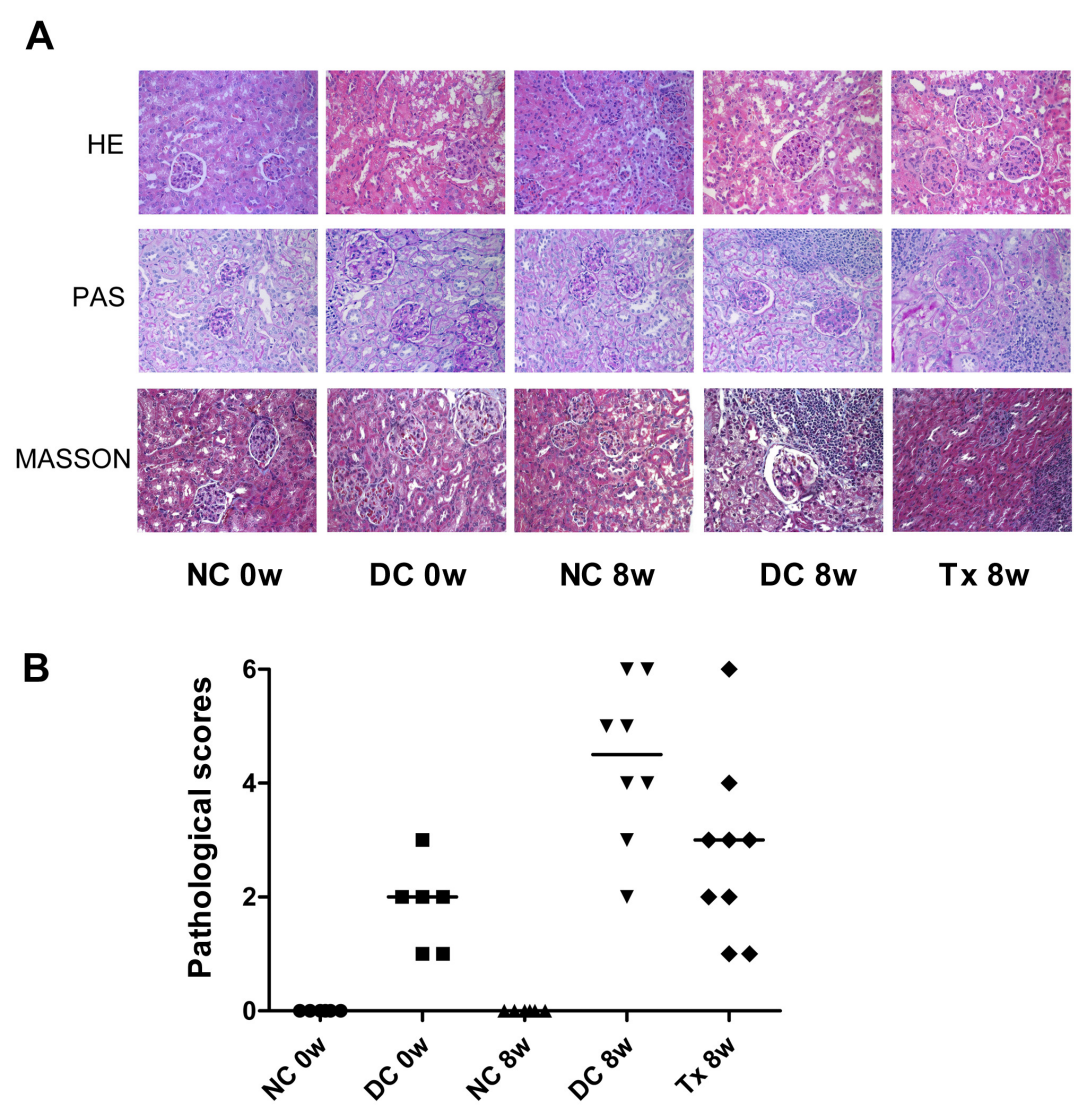
TAC attenuated renal pathology damage in MRL/lpr mice. (A) At the end of the treatment period (8 weeks), kidney tissues from mice (starting at 12 weeks of age) were immersion-fixed in 4% paraformaldehyde/phosphate buffered saline and embedded in paraffin. Sections (2 μm) were stained with HE, PAS and Masson stain. There were 8 and 9 mice in DC 8w and Tx 8w group, respectively; 6 per group in other groups. (B) Thirty glomeruli for each kidney section were digitally quantified. A microscope equipped with a color camera was used and data were analyzed using image analysis software. According to the glomerular, renal tubular and pathology rating criteria [5], we calculated pathological scores in each mouse. NC, normal control group; DC, disease control group; Tx, treatment group. DC 8w vs. NC 8w, *P* < 0.01; DC 8w vs. Tx 8w, *P <* 0.05.

There was no glomerular, interstitial or vascular injury in the normal control group, but these pathology characteristics became readily observable in the disease group. Cellular proliferation and/or membrane thickness in glomeruli, and interstitial inflammatory cell infiltration were observed (Fig 2A). Pathological scores indicated significant tissue damage in the disease control group whereas those not found in the normal control group. After TAC treatment, pathological scores were lower than those in the disease control group (Fig 2B).

### Tacrolimus reserved synaptopodin and stabilized podocyte cytoskeleton

**Fig 3:**
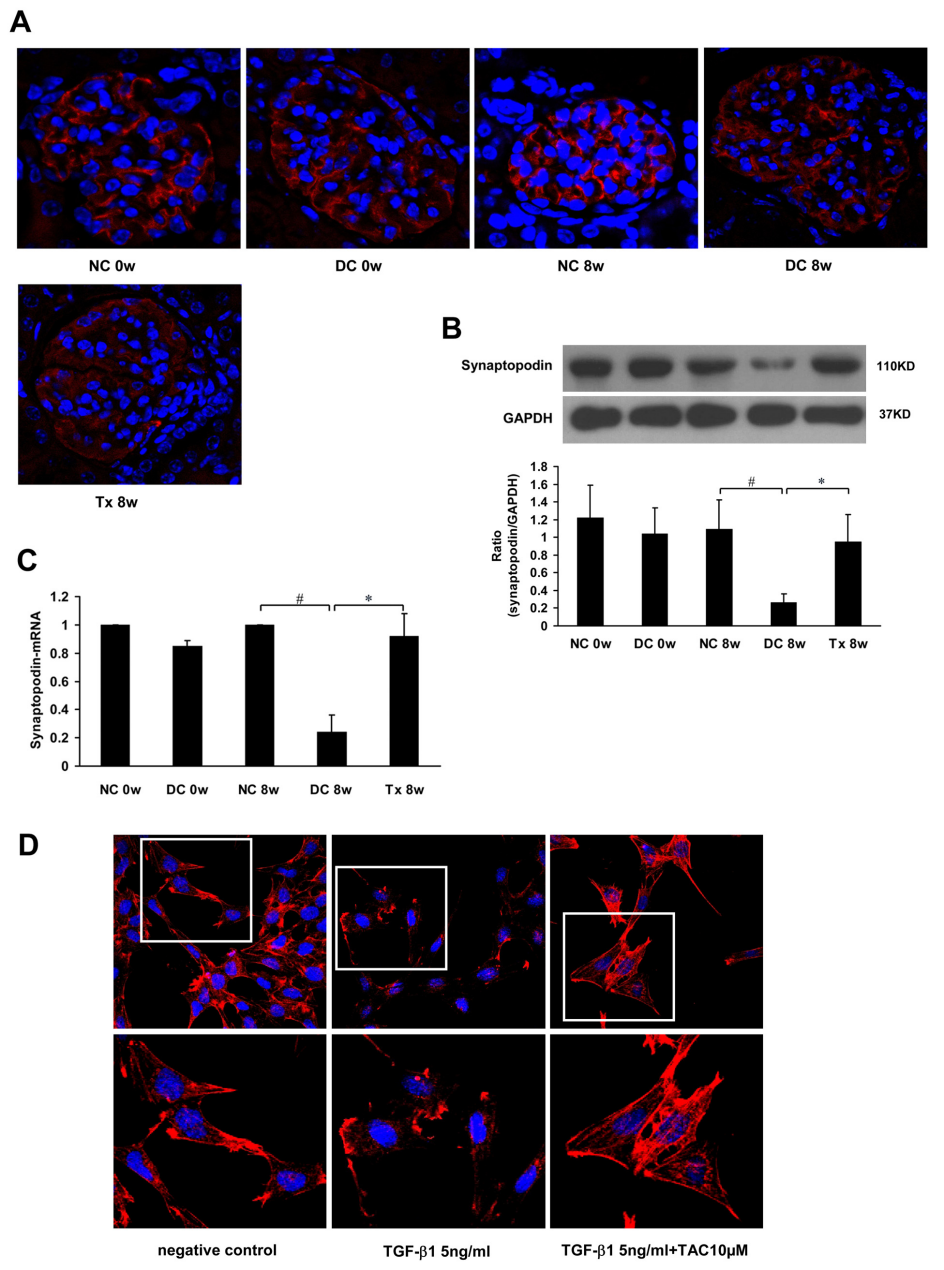
TAC restored synaptopodin expression and stabilized podocyte cytoskeleton. (A) TAC restored synaptopodin expression in MRL/lpr mice (starting at 12 weeks of age, 6 per group), as shown by immunofluorescence. (B) Western blot showing synaptopodin expression in each group (n = 6). (C) Real time-PCR showing synaptopodin mRNA in each group (n = 6). (D) Treatment with TAC attenuated the decrease in F-actin staining (phalloidin staining) induced by TGF-β1 (5ng/ml) in mouse podocytes (MPC5 cells). NC, normal control group; DC, disease control group; Tx, treatment group. * *P* < 0.05, ^#^ *P* < 0.01.

Immunofluorescence (IF) analysis revealed that synaptopodin was distributed along glomerular capillary loops in a continuous, linear form (Fig 3A). Western blot analysis showed that synaptopodin protein levels were significantly reduced at 8 weeks in the disease control group and markedly recovered after treatment for 8 weeks (Fig 3B). Real time-PCR results showed that synaptopodin mRNA declined in the disease control group, but significantly increased after 8 weeks of treatment (Fig 3C).

In the normal control group, the cytoskeleton from MPC5 cells was continuous and showed a filamentous arrangement. In groups treated with 5 ng TGF-β_1_/ml, the MPC5 cytoskeleton was granular and its integrity was damaged. In contrast, the cytoskeleton was stable and its integrity was restored in groups treated with 5 ng TGF-β_1_/ml + 10 μM TAC (Fig 3D).

### Tacrolimus preserved podocyte and reduced podocyte apoptosis

**Fig 4:**
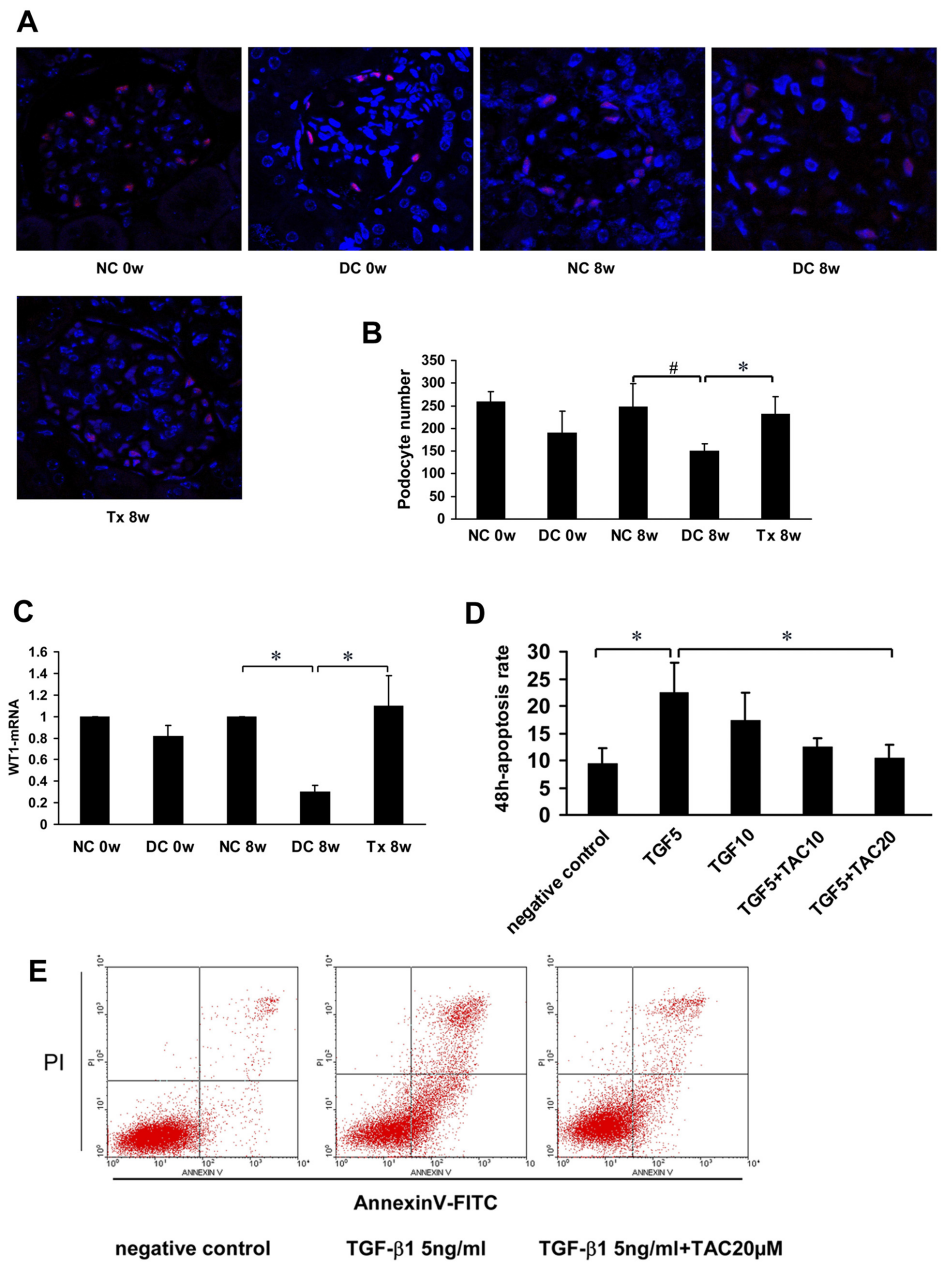
TAC preserved podocyte number and reduced podocyte apoptosis. (A) WT1 expression in MRL/lpr mice (starting at 12 weeks of age, 6 per group), as shown by immunoflourescence. (B) Podocyte number per mean glomerular tuft in each group (n = 6). (C) Real time-PCR analysis of WT1-mRNA expression was performed, n = 4. (D, E) Mouse podocyte cells were pre-incubated with TAC (10 μM or 20 μM) followed by treatment with TGF-β_1_ (5 ng/ml or 10 ng/ml); n = 4. Apoptosis was assessed with annexin V by flow cytometery (FCM). TGF5, TGF-β_1_ 5 ng/ml; TGF10, TGF-β_1_ 10 ng/ml; TAC10, TAC 10 μM; TAC20, TAC 20 μM. NC, normal control group; DC, disease control group; Tx, treatment group. * *P* < 0.05, ^#^ *P* < 0.01.

Immunofluorescence analysis showed that WT1 was specifically located in podocyte nuclei and the number of podocytes was significantly decreased in the disease control group and gradually increased after TAC treatment (Fig 4A and 4B). Real time-PCR results showed that WT1 mRNA gradually decreased in the disease control group and gradually increased after treatment (Fig 4C). In groups treated with TGF-β_1_, apoptosis rates increased to varying degrees and decreased in the groups treated with TGF-β_1_ + TAC (Fig 4D). Among them, there were statistically significant differences between the 5 ng TGF-β_1_/ml group and the 5 ng TGF-β_1_/ml + 20 μM TAC group (Fig 4E).

### Tacrolimus inhibited foot process fusion in MRL/lpr mice

**Fig 5:**
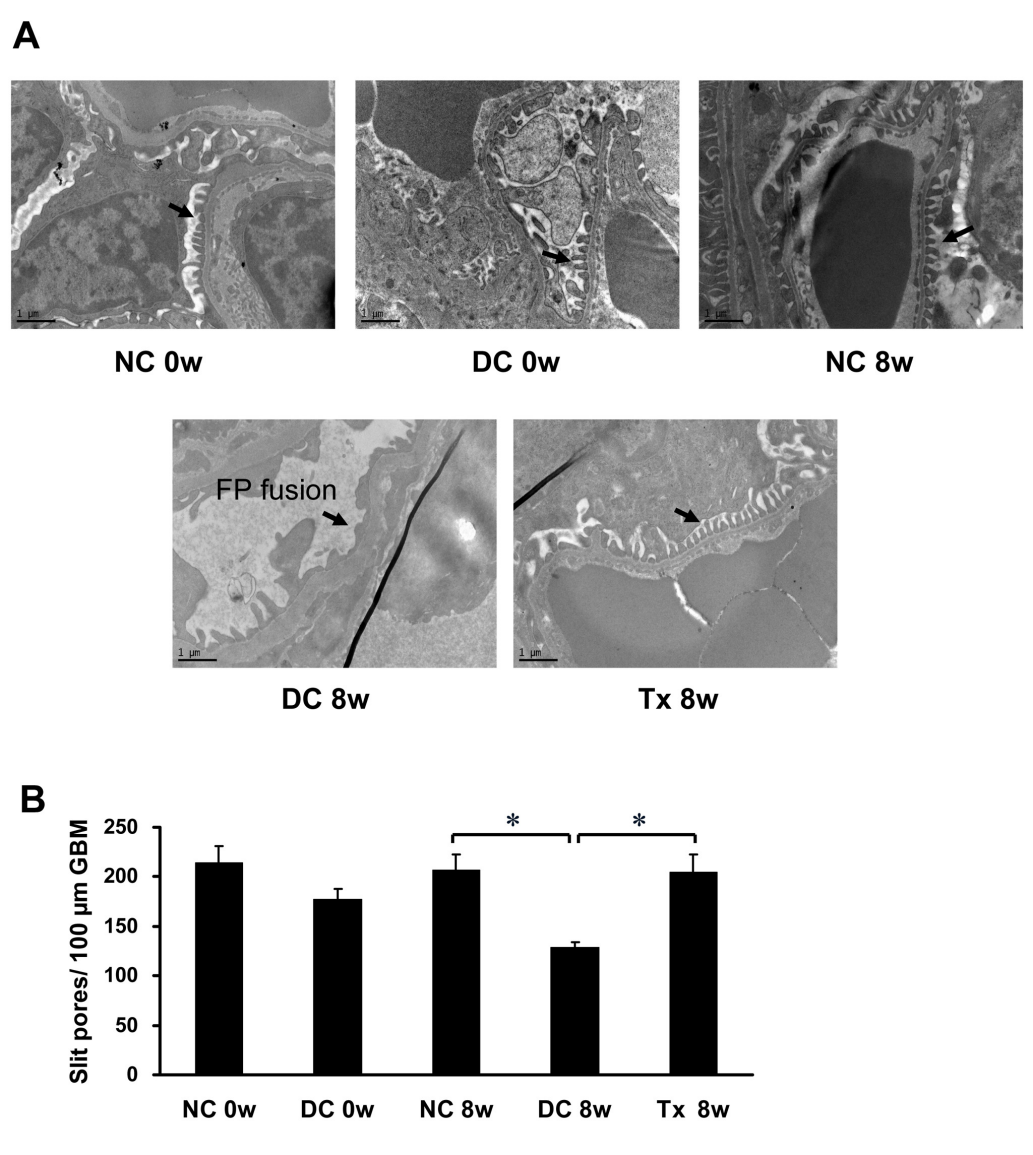
TAC prevented the effacement of foot process in MRL/lpr mice. (A) TAC prevented the effacement of foot process (arrows) and preserved the slit diaphragm in MRL/lpr mice (starting at 12 weeks of age, 6 per group), as shown by transmission electronic microscopy (13,500×). (B) The number of slit pores per 100 μm length of glomerular basement membrane (GBM). FP, foot process. NC, normal control group; DC, disease control group; Tx, treatment group. * *P* < 0.01.

Normal control mice showed a relatively evenly spread slit diaphragm. In contrast, in the disease control group and before treatment, podocyte foot process structure disappeared from view and appeared to exhibit partial or complete fusion indicative of the decreased open slit pores. After TAC treatment, the foot processes recovered and had a "zipper-like" appearance (Fig 5A), associated with a significant increase in the number of slit pores (Fig 5B).

### Tacrolimus suppressed glomerular immune complex deposition in MRL/lpr mice

**Fig 6:**
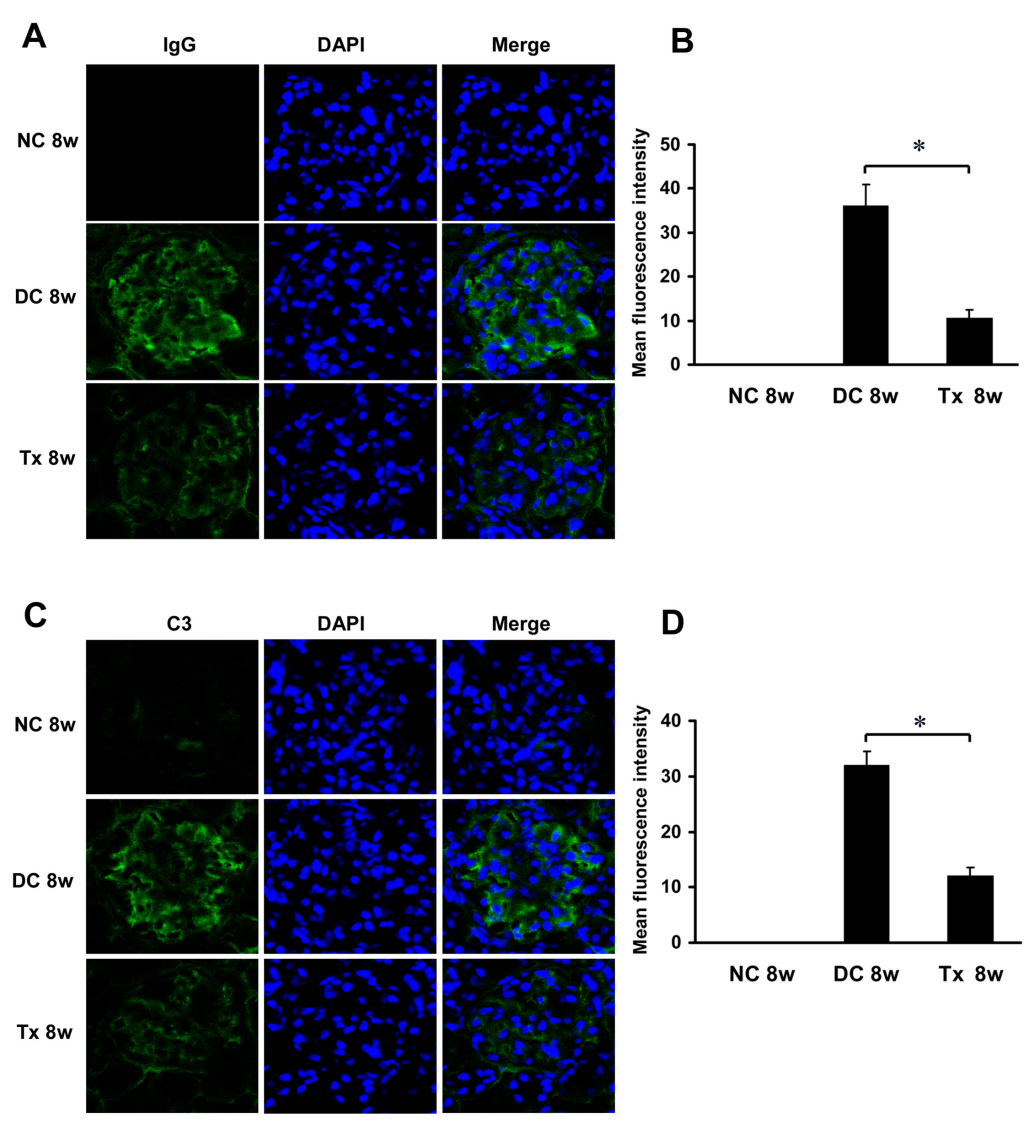
TAC reduced Glomerular IgG and C3 deposition in MRL/lpr mice. (A) TAC administration decreases IgG deposition in glomeruli of MRL/lpr mice (starting at 12 weeks of age, 6 per group), as shown by immunoflourescence. (B) The mean fluorescence intensity corresponding to IgG staining/deposition (n = 6). (C) TAC treatment decreases C3 deposition in glomeruli. (D) The mean fluorescence intensity corresponding to C3 staining/deposition (n = 6). Magnification, ×400 in A and C. Nuclei are counterstained with DAPI. NC, normal control group; DC, disease control group; Tx, treatment group. * *P* < 0.05.

Immunofluorescence analysis showed that there was no glomerular IgG deposition in the normal control group. And large amounts of IgG deposition can be observed in glomeruli of the disease control mice, while in contrast, TAC treatment significantly limited deposition of IgG (Fig 6A and 6B). Similarly, C3 staining in the kidney sections revealed decreased deposition in the TAC-treated mice as compared to the disease control animals (Fig 6C and 6D).

## Discussion

In current work, we demonstrated that TAC treatment results in a reduction of proteinuria and significant improvement in renal function in MRL/lpr mice. The results of histopathological examination also showed that TAC treatment attenuates renal pathology. A recent review showed that proteinuria can result from podocyte injury [8]. We, therefore, also examined the effect of TAC on podocyte number and actin cytoskeleton. Our data showed that TAC reserved synaptopodin expression and stabilized podocyte cytoskeleton, and also preserved podocyte number, reduced podocyte apoptosis and inhibited foot process fusion, suggesting that TAC can maintain the integrity of podocytes and glomerular filtration barrier and thereby reduce proteinuria.

TAC, which is a relatively new CNI isolated in 1984 [9], is often recognized for its immunosuppressive properties and administrated extensively in transplantation in the recent years. Li *et al* [10] conducted a single-center prospective study and found that TAC can quickly and effectively induce relief from resistance to steroids and cyclophosphamide in adult nephrotic syndrome, improve serum albumin levels, and maintain renal function. Early animal experiments found that TAC could significantly reduce BUN, proteinuria and dsDNA levels, and improve kidney damage in lupus mice [11]. In this investigation, our study results confirmed that TAC can effectively reduce 24-h proteinuria in MRL/lpr mice, effectively improve renal function and pathological damage.

It has been reported that podocytes may be affected at early stages of LN and correlate with disease histology [12]. Kidney podocytes have a complex cellular organization consisting of cell body, major processes, and foot processes. The foot processes plays a prominent role in establishing the final barrier to urinary protein loss. Synaptopodin is an actin filament-associated protein and is expressed highly in podocyte foot processes [13]. In kidney disease, therefore, changes of synaptopodin may alter the structure and function of foot processes. Srivastava *et al* [14] found that synaptopodin expression was lower in kidney tissues of patients with minimal change disease (MCD), mesangioproliferative glomerulonephritis (MsPGN), or focal segmental glomerulosclerosis, as compared to those in normal patients. Similarly, our results indicated that synaptopodin expression decreased significantly in MRL/lpr disease control mice, accompanied by increases of 24-h proteinuria, BUN, and serum creatinine. We speculate that decreases of synaptopodin in lupus mice may cause ultrastructural changes of foot processes, which disturb their function, eventually leading to fusion of foot processes and their disappearance. This was confirmed by electronic microscopy in our results, showing that ultrastructural changes in foot processes correlated with proteinuria and cytoskeleton damage.

Interestingly, Faul C *et al* [4] reported that CsA, another common CNI drug, leads to a stabilization of the actin cytoskeleton and stress fibres in kidney podocytes, suggesting that the antiproteinuric effect of CsA is independent of its immunosuppressive effect and instead results from a direct effect on synaptopodin. This is consistent with our findings that after TAC therapy, the expression of synaptopodin protein and mRNA is restored, foot process fusion is clearly inhibited, and the slit diaphragm recovers. Furthermore, in an *in vitro* model, we used TGF-β_1_ to stimulate the MPC5 cells. TGF-β is a crucial mediator of cell signaling networks that control various cellular processes, such as cell differentiation and apoptosis. Increased glomerular TGF-β was found locally overexpressed in samples of LN patients [15, 16] and MRL/lpr mice [17]. It has been confirmed that TGF-β can induce podocyte injury [18]. In the present study, we observed TGF-β_1_ could damage the F-actin cytoskeleton, while the cytoskeleton recovered and stabilized after treatment with TAC. Despite not knowing the exact molecular mechanisms by which TAC protects from TGF-β-induced podocyte injury, our results further proved that TAC reduced proteinuria through protecting potocytes and stabilizing their actin cytoskeleton.

WT1 is a type of zinc-finger transcription factor located in the nucleus of mature podocytes [19], and is considered a marker of podocytes [20]. The loss of podocytes is observed in severe kidney diseases, including IgA nephropathy, LN and diabetic nephropathy [21]. Our results showed that the expression of WT1 was reduced in MRL/lpr mice, accompanied by an increase in proteinuria. After TAC treatment, however, WT1 expression increased, which suggests that TAC can increase podocyte numbers in MRL/lpr mice.

Apoptosis is a type of the programmed cell death controlled by genetic factors [22] and has an important role in the regulation of cell number, quality and maintenance of homeostasis. Inhibition of apoptosis is usually required to promote cell survival [23]. Recently it has been reported that TGF-β_1_ can greatly induce apoptosis in cultured mouse podocytes [24]. In this work, we found that TAC significantly suppressed TGF-β_1_ induced apoptosis in MPC5 cells *in vitro*. Our data are consistent with previous findings that CsA can inhibit apoptosis of human endothelial cells [25] and myeloid leukaemia cells [26]. However, these data argue against the evidence published that CsA induced apoptosis in dose- and time-dependent manners in a permanent podocyte cell line [27]. Recently, it was reported that the proapoptotic effect of CsA could not be reproduced in various murine podocyte cell lines [28]. The effects of CsA on apoptosis are controversial, with questions existing about the origin of the cell lines used in some studies.

In addition, it is well known that deposition of immune complexes in glomeruli is a characteristic feature in LN. Podocytes damage mediated by immune complexes was involved in lupus nephritis [29]. TAC, which is known to inhibit T cell immunity, can also inhibit B-cell activation through interfering with interactions between T cells and other cells [30, 31]. We therefore further investigated whether TAC treatment could block IgG and C3 deposition in glomeruli in MRL/lpr mice. Our results showed glomerular deposition of these immune complexes was significantly inhibited in the TAC-treated group, suggesting that TAC probably also exert its protective effects on podocytes through decreasing the deposition of immune complexes in glomeruli. Our data are consistent with previous findings that treatment with TAC significantly reduced glomerular deposition of C3 in MRL/l mice [11]. Quinn et al [32] has also obtained similar results that CsA treatment results in inhibition or diminution of IgG and C3 deposition in an antigen-induced GN model. The precise mechanisms of the immune complexes injuring podocytes deserve further investigation in LN.

In summary, our data indicate that in addition to attenuating the glomerular deposition of immune complexes, the anti-albuminuria and renal protective effects of TAC may partly result from stabilizing the actin cytoskeleton and maintaining podocyte number, which, in turn, are effective to preserve foot process and maintain the blood–urine barrier and thereby improve proteinuria and kidney function in LN. These findings may provide new insights into understanding that TAC, in addition to its immunosuppressive effect, can influence the structure and function of podocytes, suggesting that the drug that directly preserves podocytes may prove to be a novel therapeutic agent for LN.
